# Distinguishing Childhood Asthma Exacerbations from Stable Asthma: The Utility of Inflammatory White Blood Cell Biomarkers

**DOI:** 10.3390/diagnostics14151663

**Published:** 2024-08-01

**Authors:** Ali Alsuheel Asseri

**Affiliations:** Department of Child Health, King Khalid University, Abha 62529, Saudi Arabia; alsoheel11@kku.edu.sa; Tel.: +966-500186013; Fax: +966-2418139

**Keywords:** childhood asthma, neutrophil-to-lymphocyte ratio, eosinophil, asthma exacerbation, systemic inflammation

## Abstract

Background: Asthma is a chronic inflammatory condition characterized by episodes of acute asthma exacerbations (AAEs), in addition to chronic airway inflammation, which has a huge impact on both the affected patients and their parents. The main objective of this study was to explore the utility of available white-blood-cell-derived inflammatory markers in diagnosing AAEs and identifying children at risk for severe exacerbations requiring admission to the pediatric intensive care unit (PICU). Methods: This study was a retrospective cohort study. The medical records of 128 children diagnosed with asthma exacerbation and 131 children with stable asthma between the ages of 2 and 12 years were reviewed. Results: A total of 259 participants were enrolled. Children with AAE demonstrated significantly higher white blood cell counts (WBC: 10.0 ± 4.2 × 10^3^/μL vs. 7.1 ± 2.2 × 10^3^/μL, *p* < 0.001), absolute neutrophil counts (ANC: 7398.5 ± 4600 cells/μL vs. 2634.8 ± 1448 cells/μL, *p* < 0.001), and neutrophil-to-lymphocyte ratios (NLR: 7.0 ± 6.8 vs. 0.9 ± 0.7, *p* < 0.001) but significantly lower absolute lymphocyte counts (ALC: 1794.1 ± 1536 × 10^3^/μL vs. 3552.9 ± 1509 × 10^3^/μL, *p* < 0.001). Interestingly, blood eosinophil count displayed an opposite trend: children with stable asthma had significantly more eosinophils compared to those experiencing an exacerbation (370.1 ± 342.7 cells/mm^3^ vs. 0.9 ± 1.9 cells/mm^3^, *p* < 0.001). Two criteria that are indicative of AAE were identified: NLR values greater than 1.2, with good discriminative ability (area under the curve [AUC] 0.90; 95% confidence interval [CI] 0.85–0.94; sensitivity 82.5%; specificity 79.5%), and ANC values exceeding 3866, with moderate discriminative ability (AUC 0.86; 95% CI 0.81–0.91; sensitivity 75.0%; specificity 82.3%). Moreover, a comparative analysis of these markers (NLR, ANC, PLR, WBC, AEC, and ALC) in patients with AAE did not demonstrate significant differences between those requiring PICU admission and those who did not require it. Conclusions: This study contributes two major findings. The first is that NLR, ANC, WBC, and PLR are significantly higher in AAE patients compared to those with stable asthma. The second is that children with stable asthma have higher AEC and ALC levels compared to those with AAE. Furthermore, this study has revealed that the studied markers (NLR, ANC, PLR, WBC, AEC, and ALC) did not differentiate between AAE patients requiring PICU admission and those managed in the general ward, suggesting a need for alternative predictive factors.

## 1. Introduction

Asthma is a chronic inflammatory condition characterized by episodes of acute asthma exacerbations (AAEs), in addition to chronic airway inflammation, which has a huge impact on both the affected patients and their parents [1,2]. This burden is characterized by a high rate of emergency department (ED) visits and hospital admissions; furthermore, its detrimental effects reach into the socio-educational domain, in addition to increasing asthma morbidity and healthcare costs. In particular, AAEs may cause parents to miss workdays, increase the requirement for special education interventions, increase school absenteeism, and result in worse academic performance, as reflected by lower exam scores [1,3,4]. In the United Kingdom and the United States of America, estimates based on prevalence data suggest that asthma affects over 1 million and 5 million children, respectively, solidifying its position as the most prevalent chronic disease in the pediatric population [5].

Pediatric AAE is typically characterized by the acute or subacute progressive worsening of asthma symptoms, namely respiratory distress and airflow obstruction, that could be either the first presentation of asthma or occur after a period of asthma control, necessitating unscheduled ED visits with the initiation of systemic corticosteroids and bronchodilator therapy. The spectrum of severity in these episodes is wide, ranging from mild to life-threatening. Furthermore, each exacerbation carries a risk of progression to respiratory failure and admission to intensive care [6,7]. The current standard for diagnosing AAEs is largely subjective and relies primarily on clinical evaluation, including patient history, physical examination, and, rarely, a pulmonary function test [7,8,9]. While these methods are valuable, they have limitations. History-taking can be unreliable, especially in young children, and spirometry may not be feasible during an acute episode due to a lack of patient cooperation or respiratory distress. Additionally, existing tools for risk stratification, such as clinical scoring systems, have shown limited accuracy in predicting exacerbation severity and the need for intensive care admission. Therefore, there is a critical need for the development of novel and more objective diagnostic tools to improve the diagnosis and management of AAEs in children.

While the precise etiology of AAE remains unknown, respiratory viral infections are frequently recognized as triggering factors in established asthmatic individuals with type 2 inflammatory airway disease. Notably, AAE severity exhibits a direct correlation with the underlying chronic inflammatory state of the airways [10,11]. While medium-sized airways are the primary site of pathophysiology in asthma exacerbations, the inflammatory process can encompass all components of the respiratory system, including the nose and the large airways [12,13]. The timely identification of inflammation would allow for significant advances in predicting and potentially preventing asthma exacerbations through early intervention and the mitigation of airway remodeling [14,15]. To date, several indirect markers have been identified that indicate the type and severity of airway inflammation even in the absence of asthma symptoms, particularly in the stable state. Specifically, the techniques for assessing asthma severity and phenotype/endotype include spirometry, peripheral eosinophilia, exhaled nitric oxide, and allergen sensitization [14]. However, despite these measures being utilized in asthma phenotyping and the prediction of AAE, their role in AAE assessment remains limited [14,16,17]. The identification of novel biomarkers for the diagnosis and assessment of AAE, therefore, represents a critical unmet need. 

Several inflammatory markers, such as the white blood cell (WBC) count, neutrophil-to-lymphocyte ratio (NLR), and eosinophil count, have recently shown promise in differentiating between AAE and stable asthma [18,19]. We speculate that these parameters do, indeed, differ between children with AAEs and children with stable asthma. Therefore, the main objective of this study was to explore the utility of available white-blood-cell-derived inflammatory markers in diagnosing AAEs and identifying children at risk for severe exacerbations requiring admission to the pediatric intensive care unit (PICU). We anticipate that understanding the diagnostic utility of these parameters among pediatric patients with AAEs will help in their early diagnosis and, subsequently, the prompt initiation of therapies.

## 2. Materials and Methods

### 2.1. Setting and Design

This study was conducted at Abha Maternity and Children Hospital, located in Abha, Saudi Arabia. This tertiary care hospital provides comprehensive pediatric care, including a dedicated department for pulmonology and a well-equipped emergency department. This study was a retrospective cohort study. We reviewed the medical records of 128 children diagnosed with asthma exacerbation and 131 children with stable asthma between the ages of 2 and 12 years who presented to Abha Maternity and Children Hospital between January 2021 and December 2021.

### 2.2. Study Population and Data Collection

We identified two groups of children from the medical records. The AAE group comprised children aged 2–12 years with a documented diagnosis of asthma who were hospitalized with an acute exacerbation during the study period, while the stable group included children aged 2–12 years with a documented diagnosis of asthma who presented to the clinic for routine asthma management during the study period. Physician-diagnosed asthma was established based on criteria outlined by the Global Initiative for Asthma (GINA). These criteria encompass recurrent episodes characterized by shortness of breath, cough, wheezing, or chest tightness. These episodes can be triggered by various factors, including viral respiratory infections or allergen exposure. Additionally, a positive response to a two-month inhaled steroid trial is considered a key element in the diagnosis [3]. Children with diagnoses of other chronic respiratory illnesses (e.g., cystic fibrosis, congenital anomalies of the airway) or incomplete medical records were excluded. Data were collected from the electronic medical records of participants, including demographics, asthma diagnosis date, asthma severity classification, medication history, and details of the presenting symptoms and treatment during the identified asthma exacerbation (for the exacerbation group). The data collection sheet consisted of two sections: Clinical and demographic variables and laboratory variables. Clinical and demographic variables included age, gender, previous PICU admissions, admissions in the last year, and previous inhaled corticosteroid (ICS) use. The laboratory section included white blood cell counts with differentials. 

### 2.3. Statistical Analysis

The SPSS software program version 29 (IBM SPSS Statistics for Windows, Armonk, NY, USA: IBM Corp.) was used for statistical analysis. Continuous variables are presented as the mean and standard deviation (SD). Categorical variables are represented as counts and percentages. Statistical analysis of differences in categorical variables was performed using Pearson’s chi-square (χ^2^) test and Fisher’s exact test, as appropriate, and analysis of group differences was performed with Student’s *t*-test. Receiver operating characteristic (ROC) curves were generated to determine the diagnostic accuracy of these biomarkers—specifically, NLR, ANC, WBC, PLR, AEC, and ANC—classifying children with AAE and with stable asthma. Differences were considered significant when the *p*-value was less than 0.05.

## 3. Results

### 3.1. Clinical and Laboratory Characteristics of the Study Population

**Figure 1:**
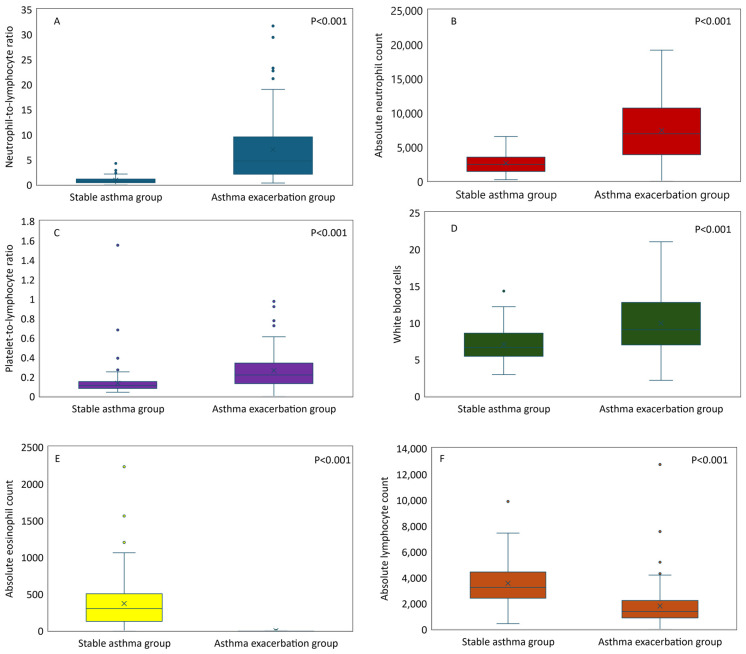
Comparison of leukocyte parameters among childhood asthma subgroups (stable, exacerbation): neutrophil-to-lymphocyte ratio (**A**), absolute neutrophil count (**B**), platelet-to lymphocyte ratio (**C**), white blood cells (**D**), absolute eosinophil count (**E**), and absolute lymphocyte count (**F**). All comparisons are statistically significant (*p* < 0.05).

Table 1 presents the clinical and epidemiological characteristics and laboratory findings of the enrolled patients. This study enrolled a total of 259 participants, comprising 128 children experiencing an acute asthma exacerbation and a comparison group of 131 children with stable asthma. The mean age did not differ significantly between the groups (*p* = 0.214). Children with AAE had a mean age of 5.32 years (SD ± 2.5), while those with stable asthma had a mean age of 5.12 years (SD ± 1.2). In contrast, a statistically significant difference (*p* < 0.001) was observed in the sex distribution. The stable asthma group exhibited a male predominance, with males comprising 64% of the group. A statistically significant difference (*p* = 0.006) was also observed in the frequency of previous PICU admissions. Children with stable asthma had a higher rate of prior PICU admission (*n* = 30, 23.0%) compared to those with AAE (*n* = 22, 17.2%). Similarly, hospitalization rates in the previous year differed significantly between the groups (*p* = 0.008). Children in the exacerbation group had a higher hospitalization rate (*n* = 63, 49.2%) compared to those in the stable asthma group (*n* = 59, 45.0%). Several lymphocyte parameters also differed. Children with AAE demonstrated significantly higher white blood cell counts (WBC: 10.0 ± 4.2 × 10^3^/μL vs. 7.1 ± 2.2 × 10^3^/μL, *p* < 0.001), absolute neutrophil counts (ANC: 7398.5 ± 4600 cells/μL vs. 2634.8 ± 1448 cells/μL, *p* < 0.001), neutrophil-to-lymphocyte ratios (NLR: 7.0 ± 6.8 vs. 0.9 ± 0.7, *p* < 0.001), and platelet-to-lymphocyte ratios (PLR: 0.3 ± 0.2 vs. 0.1 ± 0.1, *p* < 0.001) but significantly lower absolute lymphocyte counts (ALC: 1794.1 ± 1536 × 10^3^/μL vs. 3552.9 ± 1509 × 10^3^/μL, *p* < 0.001). Interestingly, blood eosinophil count displayed an opposite trend: children with stable asthma had significantly more eosinophils (370.1 ± 342.7 cells/mm^3^) compared to those experiencing an exacerbation (0.9 ± 1.9 cells/mm^3^, *p* < 0.001). A significant difference was also evident in the platelet count (323.2 ± 99 × 10^3^/μL vs. 403.0 ± 517 × 10^3^/μL, *p* = 0.045), but the magnitude of the difference was small compared to the other parameters (Figure 1).

### 3.2. Inflammatory Indices in Stable Asthma vs. Asthma Exacerbations

**Figure 2:**
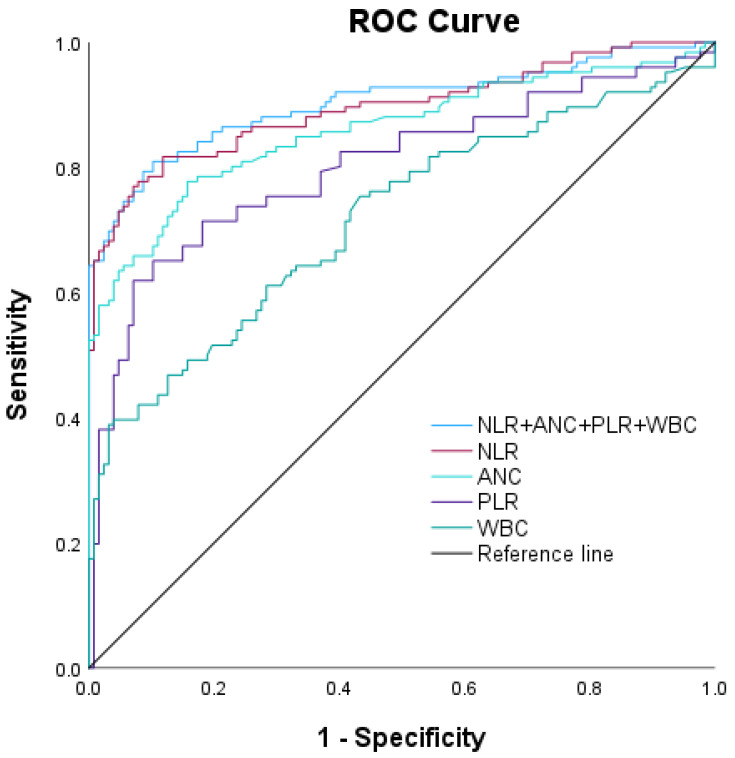
Receiver operating characteristic curves indicating the diagnostic efficacy of four leukocyte parameters for asthma exacerbation: NLR: neutrophil-to-lymphocyte ratio; ANC: absolute neutrophil count; PLR: platelet-to-lymphocyte ratio; WBC: white blood cell count.

Our analysis identified several laboratory markers that may be helpful for differentiating between AAE and stable asthma (Table 2). The first is the NLR, for which values greater than 1.2 yielded an area under the curve (AUC) of 0.90 for AAE (indicating good discriminative ability), with a 95% confidence interval of 0.85–0.94. Sensitivity was 82.5%, and specificity was 79.5%. The second was the ANC, for which values exceeding 3866 resulted in an AUC of 0.86 for AAE (moderate discriminative ability), with a confidence interval of 0.81–0.91. Sensitivity was 75.0%, and specificity was 82.3%. The third was the PLR, where values greater than 0.13 showed an AUC of 0.80 for AAE (moderate discriminative ability), with a confidence interval of 0.75–0.86. Sensitivity was 75.4%, and specificity was 70.0%. Fourth, for WBC count, values lower than 8.18 had an AUC of 0.72 for AAE (fair discriminative ability), with a confidence interval of 0.66–0.78. Sensitivity was 61.1%, and specificity was 70.1%. Finally, when combing all biomarkers, an AUC of 0.90 for AAE (good discriminative ability) was obtained, with a confidence interval of 0.86–0.94. Sensitivity was 83.3%, and specificity was 82.7% (Figure 2).

**Figure 3:**
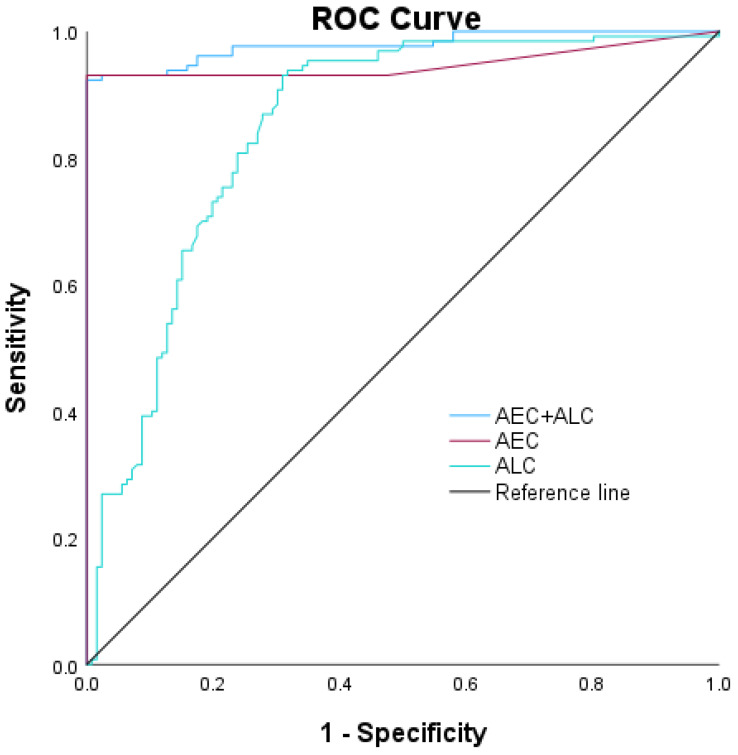
Receiver operating characteristic curves indicating the diagnostic efficacy of two leukocyte parameters for stable asthma: AEC: absolute eosinophil count; ALC: absolute lymphocyte count.

On the other hand, we also found that absolute eosinophil count (AEC) and ALC significantly discriminated stable asthma from AAE. For AEC, values greater than 10 yielded an AUC of 0.95 for stable asthma (indicating good discriminative ability), with a 95% confidence interval of 0.92–0.98. Sensitivity was 93.1%, and specificity was 100%. With ALC, values exceeding 1792 resulted in an AUC of 0.85 for stable asthma (moderate discriminative ability), with a confidence interval of 0.80–0.90. Sensitivity was 95.0%, and specificity was 67.9%. Finally, the AEC and ALC had an AUC of 0.98 for stable asthma (good discriminative ability), with a confidence interval of 0.96–0.99. Sensitivity was 96.0%, and specificity was 82.0% (Table 3 and Figure 3).

### 3.3. Inflammatory Indices in Asthma Exacerbations with PICU Admission 

**Figure 4:**
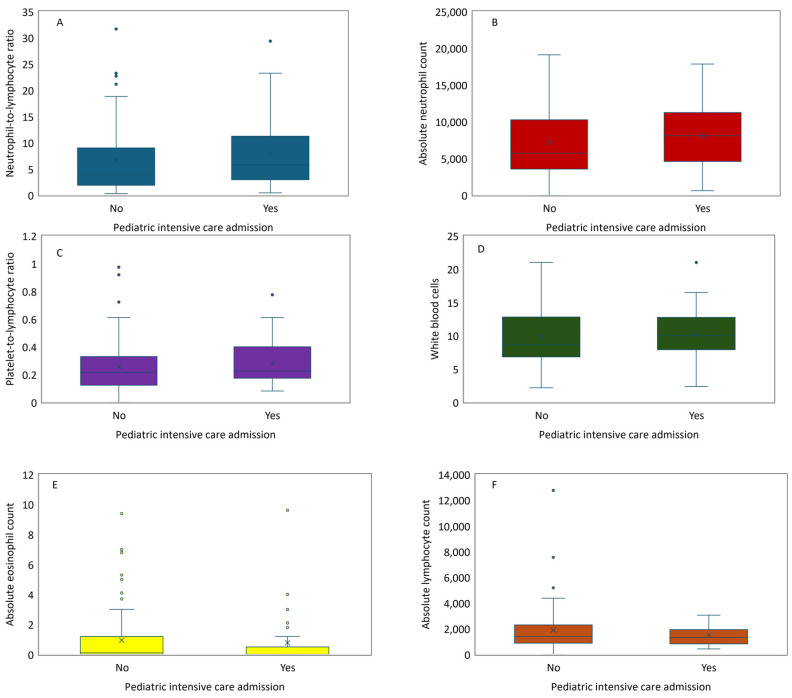
Comparison of leukocyte parameters among asthma exacerbation patients according to pediatric intensive care admission (no, yes): neutrophil-to-lymphocyte ratio (**A**), absolute neutrophil count (**B**), platelet-to-lymphocyte ratio (**C**), white blood cells (**D**), absolute eosinophil count (**E**), and absolute lymphocyte count (**F**). All comparisons are non-significant (*p* > 0.05).

The examination of six blood markers (NLR, ANC, PLR, WBC, AEC, and ALC) in children with asthma exacerbation revealed no significant differences (*p* > 0.05) in any of these markers between children who did and did not require admission to the PICU. These comparisons are summarized in Figure 4.

## 4. Discussion

This study sought to compare blood count-derived inflammatory biomarkers—specifically, NLR, ANC, WBC, PLR, AEC, and ANC—in children with AAE and with stable asthma. Prior research has emphasized the importance of these parameters in AAE, including in relation to attack severity [16,18,19]. The results of this study indicated that NLR, ANC, WBC, and PLR were significantly higher in children with AAE, while AEC and ALC were higher in children with stable asthma. Another finding that stands out from results reported previously is that these parameters could not discriminate children with AAE who required admission to PICU from those that remained in the general ward. 

Asthma is well known as a complex heterogenous disease, both phenotypically and endotypically, involving many inflammatory phenotypes [3,11]. Importantly, these background inflammatory infiltrates are dynamic and differ between children with AAE and stable asthma [20]. The presented findings demonstrated that patients with AAE have higher NLR, ANC, and WBC levels than stable asthmatics, suggesting that neutrophils may play an important role in the pathogenesis of AAE. Neutrophils are abundant circulating leukocytes that have long been recognized as the most important factor in acute bacterial infections [21]. However, recent studies have indicated AAE features’ neutrophilic predominance in airways. Norzila et al. studied airway inflammation in children with acute asthma through sputum induction and found that during acute exacerbation, the airways are characterized by the infiltration and activation of both eosinophils and neutrophils [22]. Furthermore, an autopsy of lung tissue from seven patients with fatal asthma found neutrophils to be the most abundant leukocyte [23]. In this study, the mean NLR of AAE patients was 7.0, significantly higher than that in stable asthmatics (0.9; *p* < 0.001). 

This finding is broadly supported by previous works in this area of pediatric study. Dogru et al. (2016) likewise reported a higher mean NLR (2.07) compared to healthy controls (1.77; *p* = 0.043) [24]; however, a note of caution is due here, as Dorgru et al. compared AAE patients to healthy controls, while the current study involved stable asthmatics. Nacaroglu et al. (2016) further investigated the utility of NLR as a potential inflammatory biomarker for asthma exacerbations through a longitudinal study involving 54 asthmatic children. The authors specifically compared NLR levels during exacerbation and three months later in a period of asymptomatic asthma. They reported a significantly higher mean NLR during the exacerbation period (4.9) compared to the asymptomatic period (2.4; *p* = 0.017). Notably, the mean NLR in the exacerbation group was also significantly higher compared to the healthy control group (*p* = 0.003) [25]. Meanwhile, the results of this study are contrary to those of Bedolla-Barajas et al. (2020), who found that among adult patients, NLR did not differ between those with asthma and the control group [26]. This discrepancy could be explained in part by the higher level of NLR in asthmatic children versus adults [18,27]. This finding may support the hypothesis that neutrophils play a role in airway inflammation in children with AAE. Further studies are needed that include healthy controls, in addition to stable asthmatics and AAE patients. 

Several factors may contribute to the elevated neutrophil counts observed in the current study. One potential explanation is the high concurrence of viral respiratory infections, affecting approximately 85% of the study population. Viral infections are well known to induce neutrophilia, which could elevate the overall neutrophil count [11,12,16,20,23,26]. This explanation is complicated by a report that demonstrated frequent bacterial isolation during acute wheeze attacks [10]. This study is limited by not performing invasive identification of bacterial infections, and a well-designed study will be needed to clearly answer this question. Another possible explanation is the physiological increase in neutrophils observed from neonates to adolescents [27,28]. However, this explanation is less probable due to the absence of significant age differences between the children with stable asthma and those experiencing AAE in our study. In any case, further work is needed to elucidate the exact explanation for the neutrophilic predominance, identify the underlying mechanistic pathways, and suggest therapeutic options. 

One interesting finding of the present study is the high AEC and ALC noted in stable asthmatics compared to children with AAE. These findings open a new research direction regarding the possible different inflammatory phenotypes among clinically stable asthma and asthma with AAE. Furthermore, they suggest that chronic asthma is likely characterized by underlying eosinophilic inflammation with lymphocyte activation, even in the absence of continuous allergen exposure or during periods of relatively mild symptoms. Although eosinophilic asthma is known as a common stable asthma phenotype [3], our knowledge of AAE eosinophilic phenotypes among children continues to evolve. Thus, conducting further longitudinal studies with more focus on the dynamics of inflammatory phenotypes is suggested, particularly with data collection before, during, and after asthma exacerbation episodes.

The current study has several limitations. Its retrospective design limited our ability to establish the exact baseline airway inflammatory phenotypes of patients, as well as the changes during exacerbation. Additionally, in the assessment of airway inflammation phenotypes, utilizing peripheral blood neutrophils and eosinophils as surrogate markers for airway inflammation constitutes a significant limitation. Therefore, using bronchoalveolar lavage or induced sputum cell count sampling techniques could allow for more precise phenotyping. Furthermore, as steroids inhibit neutrophil adhesion to endothelial cells and demarginate neutrophils from the marginal pool of blood vessels [29], resulting in neutrophilic leukocytosis, the lack of information regarding pre-admission steroid use would have had an impact on the prevalence of neutrophilic percentage. Finally, selection bias may arise from the inclusion of stable asthmatics without inflammatory phenotype-based classification. Future research would benefit from applying random-sample approaches among a large group of chronic asthmatics to reduce this potential bias.

## 5. Conclusions

The present study was designed to determine the diagnostic utility of inflammatory markers derived from white blood cell counts among children with AAE vs. stable asthma and identify children at risk for severe exacerbations requiring admission to the PICU. This study identified that NLR, ANC, WBC, and PLR are significantly higher in AAE patients compared to those with stable asthma. The second major finding was that children with stable asthma have higher AEC and ALC levels compared to the AAE group. These findings contribute to the current understanding of systemic inflammation among children with AAE and those with stable asthma. However, further longitudinal studies need to be carried out in order to determine the types of systemic inflammation occurring during stable asthma periods, as well as during AAE. Furthermore, this study has revealed that the studied markers (NLR, ANC, PLR, WBC, AEC, and ALC) did not differ between AAE patients requiring PICU admission and those managed in the general ward, suggesting a need for alternative predictive factors.

## Figures and Tables

**Table 1:** Clinical and laboratory characteristics of the study population.

Characteristic	Asthma Exacerbation Group (*n* = 128)	Stable Asthma Group (*n* = 131)	*p*-Value
Age, mean ± SD	5.32 ± 2.5	5.12 ± 1.2	0.214
Sex, male, *n* (%)	47 (37)	84 (64)	<0.001
Previous PICU admission, *n* (%)	22 (17.2)	30 (23.0)	0.006
Hospitalization last year, *n* (%)	63 (49.2)	59 (45.0)	0.008
ICS use ever, *n* (%)	62 (48.4)	74 (56.5)	0.107
WBC (ref: 4.3–11.0 × 10^3^ cells/μL), mean ± SD	10.0 ± 4.2	7.1 ± 2.2	<0.001
ANC (ref: 1500–8500 cells/μL), mean ± SD	7398.5 ± 4600	2634.8 ± 1448	<0.001
ALC (ref: 970–3960 cells/μL), mean ± SD	1794.1 ± 1536	3552.9 ± 1509	<0.001
Platelets, ×1000/mm^3^, mean ± SD	323.2 ± 99	403.0 ± 517	0.045
NLR, mean ± SD	7.0 ± 6.8	0.9 ± 0.7	<0.001
PLR, mean ± SD	0.3 ± 0.2	0.1 ± 0.1	<0.001
Blood eosinophil count, cells/mm^3^, mean ± SD	0.9 ± 1.9	370.1 ± 342.7	<0.001
PRAM, mean ± SD	7.1 ± 1.7	-	-

PICU: pediatric intensive care unit; ICS: inhaled corticosteroid; WBC: white blood cell count; ANC: absolute neutrophil count; ALC: absolute lymphocyte count; NLR: neutrophil-to-lymphocyte ratio; PLR: platelet-to-lymphocyte ratio; PRAM: Pediatric Respiratory Assessment Measure. *p*-value < 0.05 statistically significant.

**Table 2:** Receiver operating characteristic curve analysis results indicating the diagnostic efficacy of four leukocyte parameters for asthma exacerbation.

Variable	Criterion	AUC	AUC 95% CI	Sensitivity	Specificity	*p*
NLR	>1.2	0.90	0.85–0.94	82.5	79.5	0.000
ANC	>3866	0.86	0.81–0.91	75.0	82.3	0.000
PLR	>0.13	0.80	0.75–0.86	75.4	70.0	0.000
WBC	<8.18	0.72	0.66–0.78	61.1	70.1	0.000
NLR + ANC + PLR + WBC	-	0.90	0.86–0.94	83.3	82.7	0.000

AUC: area under the curve; CI: confidence interval; NLR: neutrophil-to-lymphocyte ratio; ANC: absolute neutrophil count; PLR: platelet-to-lymphocyte ratio; WBC: white blood cell count.

**Table 3:** Receiver operating characteristic curve analysis results indicating the diagnostic efficacy of two leukocyte parameters in stable asthma.

Variable	Criterion	AUC	AUC 95% CI	Sensitivity	Specificity	*p*
AEC	>10	0.95	0.92–0.98	93.1	100	0.000
ALC	>1792	0.85	0.80–0.90	95.0	67.9	0.000
AEC + ALC	-	0.98	0.96–0.99	96.0	82.0	0.000

AUC: area under the curve; CI: confidence interval; AEC: absolute eosinophil count; ALC: absolute lymphocyte count.

## Data Availability

Upon reasonable request, the corresponding author will provide the datasets used and/or analyzed during the current work.
